# Mercury Exposure in Goldsmithing: Unveiling Occupational Hazards

**DOI:** 10.7759/cureus.78252

**Published:** 2025-01-30

**Authors:** Inês Pereira, António Lamas, Inês Henriques Ferreira

**Affiliations:** 1 Internal Medicine Department, Unidade Local de Saúde de Santo António, Porto, PRT

**Keywords:** heavy metal poisoning, inorganic mercury poisoning, mercury poisoning, occupational disease, occupational exposure

## Abstract

Mercury poisoning in the workplace has long been recognized as an occupational disease for several decades. Elemental (or metallic) mercury can cause intoxication, mainly through inhalation, affecting workers in mining operations and goldsmiths engaged in gold recovery processes. We present the case of a professional goldsmith, a smoker with a history of narcolepsy and catalepsy. He experienced an occupational accident resulting in respiratory and mucocutaneous symptoms. The confirmation of the diagnosis by mercury measurements on the blood/urine prompted the start of chelation treatment, which led to favorable progression with symptom resolution. This case underscores the importance of public awareness to prevent mercury poisoning and emphasizes the need for healthcare providers to promptly recognize such occupational health risks.

## Introduction

Mercury, a heavy metal notorious for its detrimental effects on human health and the environment, poses significant risks to individuals exposed to its toxic properties. Since 1919, mercury poisoning, particularly chronic occupational exposure to mercury vapor, known as hydragyrism, has been officially recognized as an occupational hazard [1, 2]. In jewelry making, goldsmiths can be exposed to metallic or inorganic mercury vapors during the process of recovering gold from jewelry scraps through amalgamation, a technique also employed in gold mining [3]. Inhalation of mercury vapors poses a significant risk of acute mercury toxicity, which may lead to chronic sequelae [4].

Acute mercury toxicity [5] is primarily characterized by respiratory symptoms, including dyspnea, chest pain, and dry cough, along with gingivitis, oral stomatitis, a burning sensation in the mouth and throat, nausea, and vomiting, among other symptoms. Chronic exposure to lower levels of mercury can result in neurological manifestations, such as tremors, peripheral neuropathy, personality changes, and speech disturbances, collectively known as "mad hatter's disease" [6]. Other toxic substances, such as cyanide and nitric acid, are also utilized in these processes for metal purification [3].

Blood and/or urine measurements are essential to confirm the diagnosis, after clinical suspicion based on anamnesis. These measurements will determine the need for chelation therapy [7], an important intervention in managing mercury poisoning, and will also be useful for monitoring future mercury exposure.

## Case presentation

**Figure 1:**
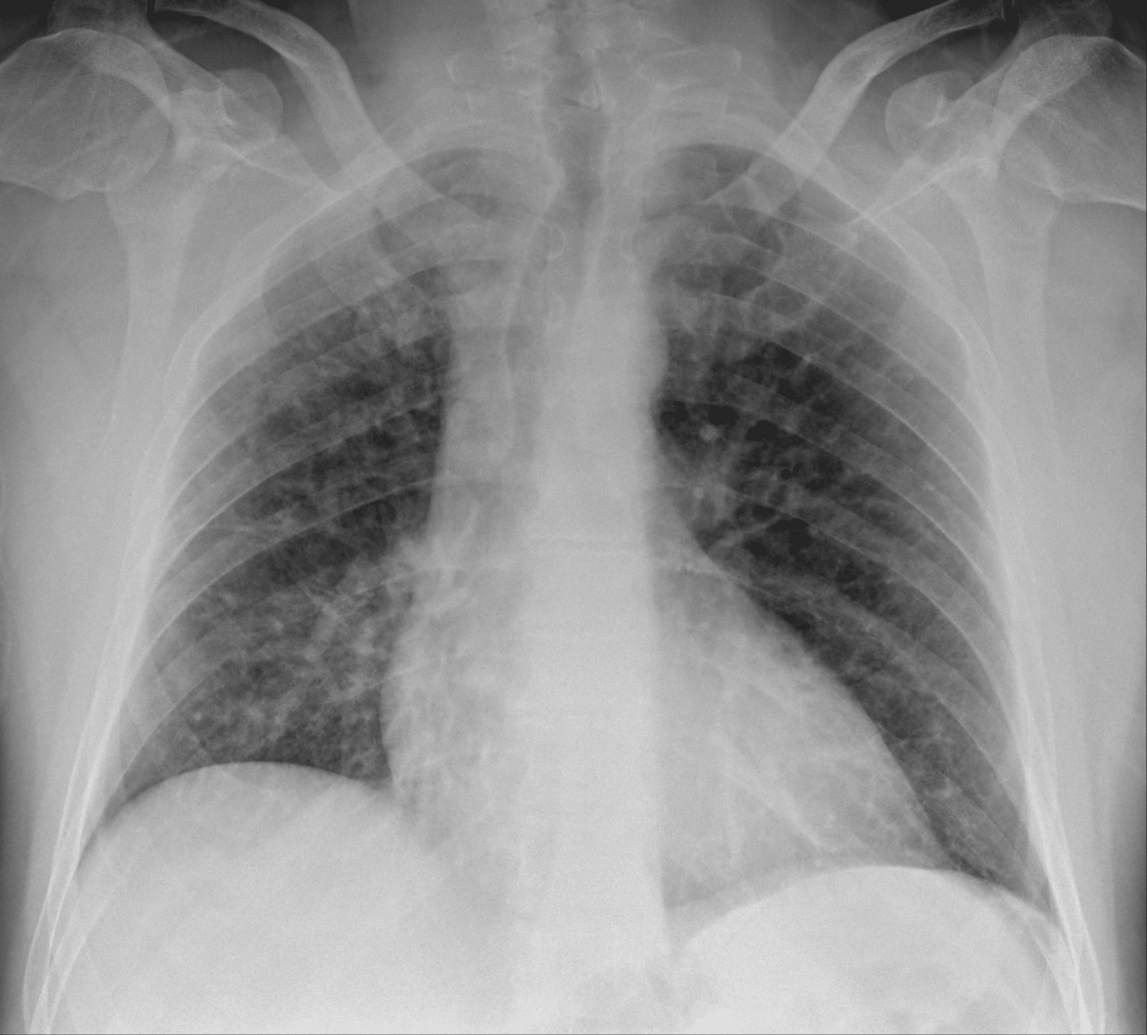
Diffuse reticulonodular infiltrates in the chest X-ray

**Figure 2:**
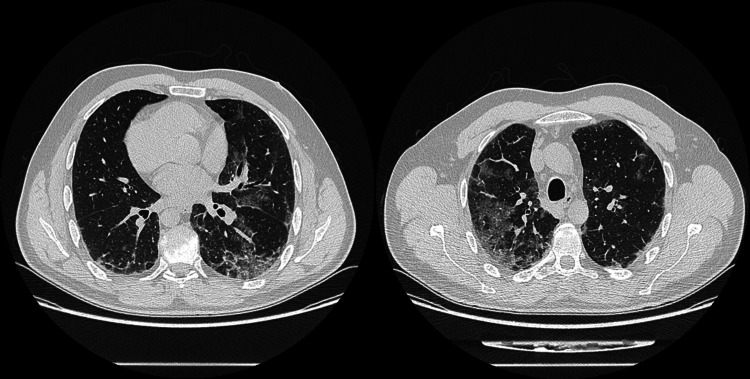
Diffuse ground-glass opacities in the chest CT

**Figure 3:**
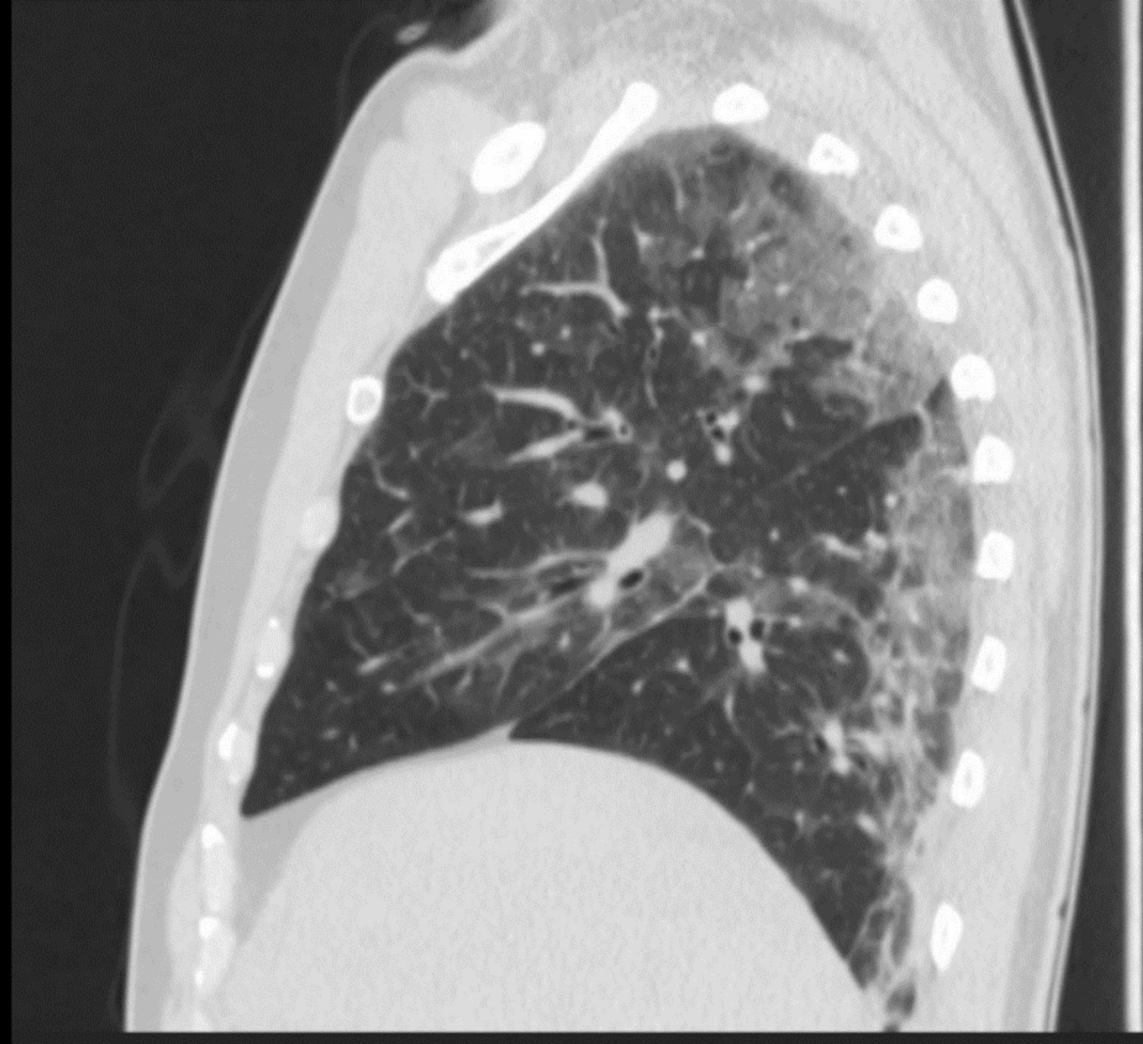
Gravity-dependent lung involvement in the chest CT

We present the case of a 49-year-old male goldsmith who is a smoker (30 pack-years) and has a history of narcolepsy and catalepsy, without any recent episodes. Four days prior to admission, he suffered an occupational accident involving exposure to elemental mercury vapor, nitric acid, and cyanide during the process of recovering gold through amalgamation. He presented to the emergency department with symptoms of fever, dyspnea, odynophagia, productive mucoid cough, and headache. Physical examination revealed angular cheilitis and signs of mucositis. Auscultation disclosed signs of bronchospasm and scattered inspiratory crackles bilaterally. Neurological examination showed no abnormalities. Laboratory blood tests indicated hypoxemic respiratory failure and elevation of inflammatory markers. No acid-base or ionic imbalances were noted, and there was no evidence of renal or hepatic dysfunction. Chest radiography revealed bilateral diffuse reticulonodular infiltrates (Figure 1), and chest computed tomography (CT) showed bilateral ground-glass densification (Figure 2), more pronounced in the right lung field and with dependent distribution (Figure 3). Supportive treatment was initiated with bronchodilators and a single dose of systemic corticosteroid (200 mg of hydrocortisone), which led to the resolution of the bronchospasm.

Mercury levels in the blood and 24-hour urine exceeded the toxicity threshold (287 µg/L and 451 µg, respectively) [8]. Following these results, chelation treatment with dimercaprol (BAL) was prescribed with the support of the hospital pharmacy, with the treatment starting at a dose of 3 mg/kg every four hours for two days. After this period, the dose was adjusted to 3 mg/kg every 6 hours for one day and then maintained every 12 hours until the subsequent assessments.

The patient experienced gradual symptomatic improvement with a resolution of respiratory failure. Monitoring of mercury levels in the 24-hour urine continued throughout the hospital stay to assess treatment efficacy. Upon discharge, the patient was advised to avoid further exposure, particularly not to engage in another amalgamation process, with no need for outpatient chelation therapy. Follow-up chest imaging at three months showed a resolution in inflammation (no ground-glass foci), the patient was asymptomatic, and mercury levels on random urine (mercury/creatinine ratio) were negative (18.0 µg/L) at six months [9]. For clarity, the laboratory results and corresponding reference values are presented in the table below (Table 1), organized by sample type.

**Table 1:** Laboratory results and corresponding reference values by sample type

Sample	Result	Reference values – toxic concentration
Blood at admission	287 µg/L	>200 µg/L
24-hour urine at admission	451 µg/24h	>50 µg/24h
Random urine	18,0 µg/L	>20 µg/L

## Discussion

Acute intoxication with metallic mercury during gold recovery processes, as seen in small-scale/artisanal gold mining, is a recognized occupational hazard [3]. Mercury toxicity varies with the form of mercury, the dose, and the rate of exposure [10]. While the fatal concentration of mercury vapor remains uncertain, clinical outcomes correlate strongly with exposure duration, mercury concentration, and survival time [5]. 

Acute intoxication by mercury vapor can lead to respiratory symptoms, consistent with chemical bronchiolitis and pneumonitis, as well as mucocutaneous manifestations, including cough, dyspnea, chest pain, inflamed gums, and excessive salivation [5]. On the other hand, chronic exposure to high concentrations of mercury can result in neurological symptoms, such as tremors, sleep disturbances, heightened anxiety, and irritability-commonly referred to as erethismus mercurialis or "mad hatter syndrome." 

The goldsmith in this case presented symptoms four days after accidental exposure to metallic mercury, a liquid at room temperature, used in an amalgam to recover gold from jewelry remnants [4]. When heated, the amalgam releases mercury vapors. While nitric acid and cyanide are also used in this process to improve efficiency [3], their toxic effects typically manifest within 24 hours or less and within seconds, respectively, presenting as acute pulmonary edema (nitric acid) [11] or metabolic acidosis (hyperlactacidemia) and severe hypoxemia (cyanide) [12]. The absence of such findings and the delayed onset of symptoms make mercury vapor the primary contributor, although nitric acid might have played a secondary role in mucosal and airway irritation. Despite descriptions of gold vapor release in industrial settings, the temperatures applied by this patient were insufficient to vaporize gold particles. Given the patient's history of narcolepsy, the possibility of aspiration pneumonia was reconsidered. However, it was ruled out after a thorough review of the clinical history and the absence of any episodes suggestive of narcolepsy. 

Mercury poisoning was confirmed via elevated mercury levels in biological samples. While blood tests are preferred for acute cases, urine samples are more suitable for chronic exposure due to mercury redistribution in tissues [13]. However, testing posed logistical challenges, as analyses were outsourced rather than performed in-house. The standard treatment involves the cessation of exposure and, in selected cases, chelation therapy. Chelation is recommended when mercury levels exceed 100 µg/L and continues until levels drop below 20 µg/L or the patient becomes asymptomatic [7]. In this case, chelation therapy with dimercaprol, the only available chelating agent at our center, was promptly initiated as the biological indicators (urine and/or blood) and clinical findings were both consistent with mercury poisoning [7]. Continuous monitoring of mercury levels is crucial for guiding the management of mercury poisoning and determining when to discontinue chelation therapy. Systemic corticosteroids were also administered, although a more robust regimen might have been beneficial, as suggested by the literature for mitigating inflammation and preventing progression to interstitial pulmonary fibrosis. Given the plausible risk of interstitial pulmonary fibrosis, a high-resolution thoracic CT scan is recommended following resolution of the acute episode.

## Conclusions

Mercury poisoning is an uncommon condition in developed countries but remains a significant occupational risk for goldsmiths involved in gold recovery processes, often due to inadequate safety measures. This case highlights the importance of maintaining a high degree of clinical suspicion, especially in regions with low awareness of these occupational exposure risks, supported by a thorough medical and occupational history, to avoid misdiagnosis with more common conditions, such as infectious diseases, which require different treatment. Recognizing the distinct features of acute versus chronic mercury poisoning and initiating prompt chelation therapy after laboratory confirmation are critical steps to prevent severe complications and improve patient outcomes.
